# Evaluating the accuracy of morphological identification of insect pests of rice crops using DNA barcoding

**DOI:** 10.1080/23802359.2018.1532334

**Published:** 2018-10-26

**Authors:** Hafiz Muhammad Tahir, Alina Noor, Sana Mehmood, Sher Muhammad Sherawat, Muhammad Akram Qazi

**Affiliations:** a Department of Zoology, Government College University, Lahore, Pakistan;; b Department of Agriculture Extension, Devis Road lahore, Pakistan;; c Punjab Agricultural Research Board, Lahore, Pakistan

**Keywords:** Agricultural pests, IPM, mt COI gene, morphometric studies, DNA barcoding

## Abstract

Accurate identification of agricultural pests is key requirement for the successful integrated pest management (IPM) program. However, due to limitations of conventional morphological methods, other molecular method like DNA barcoding is used. The current study was designed to evaluate the accuracy of morphological identification of insect pests using DNA barcoding. Morphologically, a total of 247 insect pests, representing 10 families, 18 genera, 22 species were identified. However, molecular identifications confirmed the presence of 11 families, 16 genera, and 20 species of agricultural pests. A total of 59 specimens were processed for DNA barcoding but genomic sequences of mt COI gene up to 600 bp were revived from 48 samples. Specimens that were misidentified through morphological studies were placed to their appropriate taxon, using this molecular approach. Results revealed the existence of clear barcode gap for different pest species. Moreover, the values of distance with the nearest neighbour recorded were higher than the maximum intra-sequence divergences for all species. It is concluded that DNA barcoding is a reliable technique for identification of agricultural pests, especially for immature stages when morphometric studies are ambiguous and will be helpful in the development of more effective pest management options for regulating pest species.

## Introduction

Identification of the specimens at the species level is necessary for understanding the diversity of the species, phylogenetic patterns, and evolutionary relationships (Platnick 2014). Though the insects catalog document more than one million species but millions of them are still undiscovered (Grimaldi and Engel 2005). Traditionally, different morphological traits are used for the identification of insect pests (Jinbo et al. 2011). However, identifications based on morphology are often difficult and time consuming (Barrett and Hebert 2005). Immature life stages, i.e. juveniles, early instar and pupae are not identified by routine taxonomy as most of the morphotaxonomic keys are used for the analysis of adults only (Barrett & Hebert 2005). Phenotypic plasticity often complicates the morphological identifications (Murugan et al. 2016). Cryptic species are also difficult to identify on the basis of morphology. Moreover, high level of experience is required for the effective use of taxonomic keys (Ball and Armstrong 2006).

Molecular methods are now used widely by the taxonomists to solve the intricacy associated with traditional morphological method (Navajas and Fenton 2000). Among these methods, DNA barcoding is the easiest and the most frequently used technique (Nagoshi et al. 2011; Van der Bank et al. 2012). In this technique, short standardized gene region of mitochondrial cytochrome C oxidase subunit I is used for discriminating species (Hebert et al. 2003). This specific sequence (658 bp) is known as DNA barcode and is used as a species tag or barcode tag for each animal taxa (Jinbo et al. 2011). COI gene region is preferably used as it is found in all eukaryotic life forms. Furthermore, deletions and insertions are rare in this gene region. Finally, COI fragment bears sufficient sequence divergence to discriminate the closely linked species (Hebert et al. 2003). In addition, the amplification of this COI fragment is quite easy due to its appropriate (short) sequence length and the robust universal primers (Folmer et al. 1994; Zhang and Hewitt 1997; Simmons and Weller 2001).

Sometimes, mitochondrial DNA sequences are not generated, in that case, alternate markers are used (Vences et al. 2005). These supplementary markers include ribosomal DNA internal transcribed spacers region 2 (ITS-2) (Kumar et al. 2009), 12s, 16s rDNA (Steinke et al. 2005; Vences et al. 2005; Kappner and Bieler 2006; Aliabadian et al. 2009), NADH dehyrogenase subunit 1 (nadh1) (Jalali et al. 2015) and cytochrome b (cytb) (Bradley and Baker 2001; Pfunder et al. 2004; Desalle 2006; Hajibabaei et al. 2006; Jalali et al. 2015). Unlike nuclear DNA, mitochondrial DNA evolves rapidly due to the absence of proofreading mechanisms during DNA synthesis (Hoy 2003) as these DNA sequences have no introns (Saccone et al. 1999; Floyd et al. 2009). They are preferred as mtDNA are maternally inherited and rarely undergo recombinations (Saccone et al. 1999; Birky 2001). Moreover, mtDNA are relatively abundant (generally hundreds and thousands of copies are present per cell) (Hoy 2003) so it is easy to extract mtDNA even from a small body part of the animal (Stoeckle and Hebert 2008).

The current study has been undertaken to identify the insect pests collected from the agricultural fields of Sialkot and Lahore District with the help of DNA barcoding and to evaluate the accuracy of this systematic tool for estimation of species diversity. The generated barcode sequences during present research were also compared with the previous sequences at Genebank data for confirmation of the morphometric identifications.

## Materials and methods

### Insects collection

Live insects were collected from the rice fields of Sialkot (32.494N, 74.5229E) and Lahore (31.479N, 74.2662E) District using sweeping net, visual searching, and hand picking method. Sampling was conducted from July 2017 to November 2017.

### Preservation

Insects collected in glass vials (20 mL) were brought to the laboratory in the Department of Zoology, Government College University, Lahore. Insects were washed with alcohol and transferred with the forceps to clean glass vials in the laboratory. Specimens were preserved in 95% alcohol and kept at -20 °C before DNA extraction. All the samples were labelled properly with their site of collection, collection date, collector’s name as well as Geo-coordinates of the area selected for collection.

### Morphological identification

Identification of insects to the species level was done by investigating the morphological characters of different body parts, with the help of catalogues and keys available, such as Vreden and Ahmadzabidi (1986), Pathak and Khan (1994), Chanthy et al. (2010), Gupta and Singh (2013), Murthy et al. *(*2015) and Whiting (2017). Specimens were photographed with the help of stereozoom dissecting microscope (BCVS121 &BIOCOM UK) and digital camera (Canon power shot G9 digital camera).

### DNA barcoding and sequencing

To assess the validity of morphological identification of insect pests, 658 base pairs of cytchrome C oxidase I (barcode sequences) was sequenced. For this purpose, DNA was isolated form left leg of specimens according to the protocol of Thermo Scientific "Genomic DNA Extraction Kit". Barcode gene region of specimens was amplified via PCR and PCR products were verified through agarose gel electrophoresis. For sequencing, the PCR products and tissues were sent to Canadian Centre for Biodiversity Genomics, University of Guleph, Canada. Moreover, all the sequenced specimens were stored as voucher specimens in the library of University of Guleph, Canada.

### Data analysis

Sequence evaluation tool available in the BOLD (www. Barcoding life. org) was used to compute distance summaries as well as for construction of the Neighbour-Joining tree. The analysis of Barcode gap was also performed with the help of available tool. For all species, mean and maximum intra-sequence variations as well as distance with the NN (nearest neighbour) were assessed. Barcode sequences of the sampled specimen are available online in the dataset of MTINS present in the Database of BOLD.

## Results

**Figure 1.**
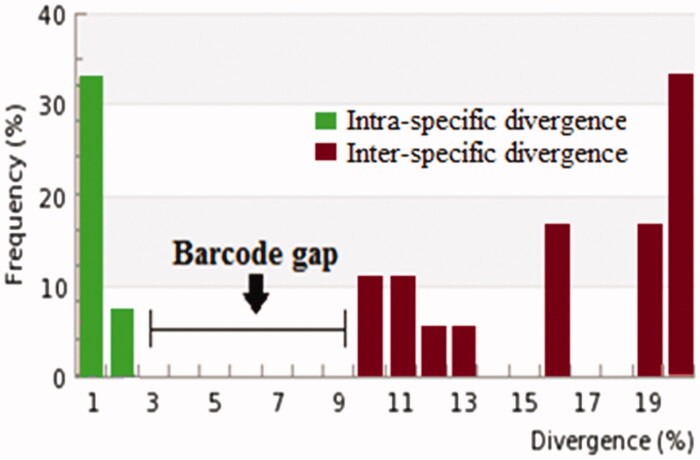
Histogram representing clear barcode gap between intra and inter-specific divergences for insect pests of different families.

Out of the total 59 specimens, genomic sequences of mt COI gene up to 600 b.p were effectively revived from 48 samples. Molecular identification studies revealed the existence of 20 species representing 16 genera and 11 families. Barcode gap of the sequenced specimens revealed that maximum values of intra-specific divergence vary from 0% in *Nilaparvata lugens, Spodoptera sp.* to 2.66% in *Atractomorpha crenulata*. The observed range of distance with their nearest neighbour (NN) was 9.28% in *Sesamia inferens, Spodoptera sp*. to 26.9% in *Scirpophaga innotata* as depicted in Table 1. Moreover, the histogram shows a clear barcode gap between intra and intersequence divergence values (Figure 1). Different species and genera were clearly separated by Neighbour-Joining tree as represented in Supplementary file 1.

**Table 1.** Mean and maximum value of intra-specific and nearest neighbour distances among different families of insect pests.

Species name	Mean intraspecific divergence	Maximum intraspecific divergence	Nearest neighbour species	Distane to NN
*Riptortus linearis*	N/A	N/A	*Cletus sp.*	18.93
*Nephotettix nigropictus*	0.06	0.15	*Nephotettix parvus*	19.46
*Nephotettix parvus*	0.15	0.31	*Nephotettix nigropictus*	19.46
*Cletus sp.*	N/A	N/A	*Riptortus linearis*	18.93
*Nilaparvata lugens*	0	0	*Scirpophaga innotata*	26.9
*Sogatella furcifera*	0.08	0.15	*Nephotettix nigropictus*	21.89
*Antestia degenera*	N/A	N/A	*Riptortus linearis*	20.92
*Cnaphalocrocis medinalis*	0.12	0.31	*Scirpophaga incertulas*	10.32
*Diaphania indica*	0.15	0.15	*Cnaphalocrocis medinalis*	11.37
*Scirpophaga incertulas*	N/A	N/A	*Cnaphalocrocis medinalis*	10.32
*Scirpophaga innotata*	0.77	0.77	*Cnaphalocrocis medinalis*	15.61
*Pelopidas mathias*	N/A	N/A	*Cnaphalocrocis medinalis*	12.81
*Sesamia inferens*	N/A	N/A	*Spodoptera sp.*	9.28
*Spodoptera sp.*	0	0	*Sesamia inferens*	9.28
*Acrida turrita*	N/A	N/A	*Oxya chinensis*	15.62
*Oxya chinensis*	0.31	0.31	*Acrida turrita*	15.62
*Atractomorpha crenulata*	1.33	2.66	*Acrida turrita*	18.93
*Conocephalus longipennis*	1.39	1.39	*Diaphania indica*	23.39

N/A denotes that species is singleton.

The retrieved barcode sequences were also judged with previous sequences in Genebank and the specimens that showed 98% similarity with other specimens in dataset were considered that particular species available in the Genebank data. These sequence comparisons revealed close resemblence (>99) for *Cnaphalocrocis medinalis*, *Diaphania indica*, *Nephotettix nigropictus*, *Nilaparvata lugens,* and *Pelopidas mathias*. However, *Sogatella furcifera* (GCUL-MTINS 26, 27) and *Riptortus linearis* (GCUL-MTINS-48) did not show any resemblance with available sequences in the Genebank revealed that these were new records to the Genebank. *Acrida sp.*, *Acrida turrita*, *Atractomorpha crenulata* showed more than 97% similarity with *Acrididae sp*., while *Atractomorpha crenulata* (GCUL-MTINS-60) showed 99% similarity with *Metaleptea brevicornis, Antestia degenera* showed 99% similarity with *Dolycoris indicus, Cletus sp.* showed 99% similarity with *Cletus pugnator, Conocephalus longipennis* showed 98% similarity with *Conocephalus maculatus, Nephotettix parvus* showed 99% similarity with *Cicadellidae sp., Nephotettix sp.* showed 99% similarity with *Nephotettix nigropictus, Oxya chinensis* showed 99% similarity with *Oxya hyla, Pelopidas mathias* (GCUL-MTINS-16) showed more than 97% similarity with *Parnara guttata, Scripophaga incertulas* showed 99% with *Spilartia oblique, Scripophaga innotata* showed 100% resemblance with *Euproctis sp, Sesamia inferens* showed 99% similarity with *Mythimna sp., Sogatella furcifera* (GCUL-MTINS-29, 30, 31, 32) showed 98% similarity with *Hemiptera sp.* and *Spodoptera sp*. showed 100% similarity with *Spodoptera litura* as shown in Supplementary file 2. Immature and misidentified specimens were placed to their appropriate taxa on the basis of their genomic sequences.

## Discussion

Morphometric identifications of the insect pests have some limitations i.e. most of the economically important pests are difficult to identify via morphotaxonomic keys even by specialists because large number of insect pests belong to morphologically cryptic species (Busvine 1980; Della Torre et al. 2002; Clark et al. 2005). Identifications of eggs and instars of pest species are difficult on the basis of morphological characters (Ball & Armstrong, 2006). In these circumstances, DNA barcoding provide quick and authentic means for species identification (Hebert et al. 2004; Ball and Armstrong 2006; Blagoev et al. 2013; Raso et al. 2014; Dona et al. 2015; Xu et al. 2015). The authenticity of DNA barcoding relies on barcode gap. Barcode gap is actually the variations in the barcode sequences, which are substantially lower in the members of same species as compared to members of closely related species; greater the barcode gap greater is the validity of results (Hebert et al. 2004; Meyer and Paulay 2005; Dasmahapatra and Mallet 2006; Meier et al. 2008). Moreover, in this technique, species is considered as distinct species from its NN (nearest neighbour), if the maximum value of intraspecific distances are less as compared to distances of its nearest neighbour (Ashfaq et al. 2014).

In the current work, morphological traits that were utilized for identification process were later confirmed via DNA barcoding. In our results, morphological method failed to recognize immature insect pests of Noctuidae family as well as some members of Crambidae, Tettigonidae, Hesperiidae, and Pentatomidae family. DNA barcoding played an important role to successfully resolve this intricacy and assigned each species to appropriate taxon. DNA barcoding is helpful to solve problems even when morphological studies are ambiguous (Candek and Kutner 2015). Many other researchers have also reported the difficulties during morphotaxonomic identifications of different insect groups that includes *Phyllaphaga sp.* larva (Coleoptera, Scarabaeidae) (Doskocil et al. 2008), *Bactrocera tryoni*, Queensland fruit fly (Tephritidae) (Blacket et al. 2012) and whiteflies (Shatters et al. 2009). DNA barcoding technique aid researchers by accurately identifying these species.

In the present study, mitochondrial COI gene based identification was successful for all pest species. At first, clear barcode gap was not observed for different pest species due to overlap in inter and intrasequence divergence values. However, when the members of *Acrida sp, Nephotettix sp, Sogatella furcifera* and *Pelopidas mathias* species that were either misidentified or showed no match with previous sequences in the Genebank data were removed, a clear cut barcode gap was observed between inter and intrasequence divergence values. Moreover, the maximum intrasequence variation recorded was lower than the distance with NN for different species of agricultural pests. Liu et al. (2014) worked on Noctuidae and Arctiidae family and observed the similar results. Similar results were attained by Gopurenko et al. (2013) during identification of leafhoppers, treehoppers, and planthoppers (Hemiptera: Auchenorrhyncha) via DNA barcoding.

In our results, Nearest Neighbour distances were effectively larger than the maximum values of intra-sequence divergences for all species. Park et al. (2011) have reported similar results for true bugs (Hemiptera: Heteroptera) i.e. less than 2% nucleotide divergence was observed within members of same species. Likewise, in tussock moth pest species, mean intra-specific barcode variations achieved was <1% (Ball and Armstrong 2006). Huang et al. (2013) reported parallel results for grasshoppers of Acridoidea family (Orthoptera: Caelifera), such as nucleotide variations within members of same species are slightly less or distinctly greater than 1%.

In some cases, barcode variations among the members of same species were exceptionally larger than previously estimated values. For example, in one of Chinese grasshopper species, *Sinopodismqa lofaoshana,* the maximum intrasequence divergences achieved was 5.56% (Huang et al. 2013). However, in our findings, the maximum value of intraspecific divergence recorded was 2.66%, which is in the range of intraspecific threshold value estimated by Carew et al. (2007) while working on various invertebrates and insect species.

It can be concluded that DNA barcoding is an effective approach for screening agricultural pests present in rice fields. In this study, morphological identification alone works satisfactorily. However, integrated barcoding, combination of traditional taxonomy and molecular methods, enhance the accuracy and reliability of results. Overall, this study contributes important information to the molecular ecology of pest species attacking paddy crops in Punjab, Pakistan, and will be helpful in the development of more effective pest management strategies.
